# Quality of albendazole tablets legally circulating in the pharmaceutical market of Addis Ababa, Ethiopia: physicochemical evaluation

**DOI:** 10.1186/s40360-019-0299-5

**Published:** 2019-04-25

**Authors:** Assegid Seifu, Elias Kebede, Belachew Bacha, Achenef Melaku, Tadese Setegn

**Affiliations:** 1 Department of Veterinary Pharmacy, College of Veterinary Medicine and Animal Sciences, University of Gondar, P. O. box= 196, Gondar, Ethiopia; 2 Animal Products, Veterinary Drug and Feed Quality Assessment Centre, Veterinary Drug and Feed Control and Administration Authority, Addis Ababa, Ethiopia; 3 Department of Food Hygiene and Technology, Faculty of Veterinary Medicine, Near East University, Nicosia, Cyprus

**Keywords:** Albendazole, FTIR, HPLC, Quality assessment, UVS, Ethiopia

## Abstract

**Background:**

Parasitic diseases are the main challenge of livestock production in the world. They are mainly controlled by the use of anthelmintic drugs. To be effective, the drugs should contain the appropriate amount of active pharmaceutical ingredient (API) and have the required physical characteristics. In this study, qualitative and quantitative assessments were performed to evaluate the quality of different brands of albendazole tablets legally circulating in pharmaceutical markets of Addis Ababa, Ethiopia.

**Methods:**

Ultraviolet–Visible Spectroscopy (UVS), Fourier Transform Infrared Spectroscopy (FTIR) and High-Performance Liquid Chromatography (HPLC) were used for identification. Quantitative analysis was performed by HPLC. United States Pharmacopeia standard was used as a control to evaluate the identity and content of the API in the samples. A total of 10 batches of albendazole tablets from six different brands were collected and evaluated.

**Results:**

All brands of albendazole tablets, except one, had acceptable physical characteristics. There was gross contamination in one batch, weight variation in 4 (40%) batches, and absence of package insert in 2 (20%) batches. All three methods of evaluation (UVS, FTIR and HPLC) confirmed that all batches passed the identity test. Quantitative analysis showed that no batch had API above the acceptable limit. However, 30% of batches from three different brands contained lower amount of API per tablet than the acceptable limit.

**Conclusions:**

All batches of albendazole circulating in the market in Addis Ababa did not fulfil either physical or chemical quality standards. The most important finding of this research was the presence of drugs with lower level of API than the acceptable limit. This can lead to treatment failure and favour the emergence of parasites that are resistant to drugs. Therefore, there should be a thorough evaluation of drugs before approval. The study also revealed the importance of occasional assessment of drugs circulating even in the legal market.

**Electronic supplementary material:**

The online version of this article (10.1186/s40360-019-0299-5) contains supplementary material, which is available to authorized users.

## Background

Parasitic diseases are the main challenge of livestock production in the world. They cause huge losses by inducing high morbidity and mortality. Hence, livestock producers use anthelmintic drugs to control helminthic parasites. Several anthelmintic drugs are available in the market. Albendazole is the most commonly used anthelmintic drug in Ethiopia [1–3]. It is used for the treatment of variety of parasitic worms due to its broad spectrums of activities [2]. To be effective in treating parasitic diseases, the drug should have the necessary physical and chemical qualities [4, 5].

Albendazole imported by private companies takes the largest share in Ethiopian market, in a limited extent the drug is also produced domestically. The quality of veterinary drugs imported, manufactured and distributed in the country is controlled by Ethiopian Veterinary Drug and Feed Control and Administration Authority. The authority has checkpoints at potential entry sites. However, there may be importation and distribution of substandard drugs [3, 6]. There are also complaints from animal health professionals and animal owners regarding the effectiveness of available drugs in the market [2]. Many stakeholders in the animal health sector have concerns about treatment failures [7].

Studies in other part of the world showed the possibility of counterfeiting on both branded and generic products [8, 9]. It has been reported that drugs on the market can have the correct ingredients, insufficient quantity of the active ingredient, wrong ingredients, no active ingredients or false or misleading packaging. They may also contain different quantities of impurities that can be harmless or toxic [10, 11].

Several studies have been conducted on the efficacy of albendazole using in vivo or in vitro techniques in Ethiopia and reported good or low efficacy [1, 3, 12]*.* The low efficacy reports may be related either to the development of drug resistance by parasites or the quality of the preparation. This necessitated further investigation and comparison with standard products. There was no attempt in the country so far to assess the quality of albendazole tablets legally circulating in the market. Therefore, this study was designed to evaluate the quality of different brands and batches of albendazole tablets sold in the legal pharmaceutical markets in Addis Ababa, Ethiopia.

## Methods

This study was conducted from November, 2016 to April, 2017 in Addis Ababa city which is the capital city of Ethiopia. The city acts as a hub for distribution of veterinary pharmaceuticals in the country. All tests were conducted in the National Animal Products, Veterinary Drug and Feed Quality Assessment Centre. Ten batches of albendazole tablets from six different brands with three different labelled dose (strength) (300, 600 and 2500 mg per tablet) of albendazole were collected randomly from different legal veterinary pharmacies in the city.

Samples were evaluated for various physical characteristics, packing information, label and information insert (leaflet), and weight. For weight evaluation, twenty tablets from each batch were weighed individually and compared [13].

For the identification test, FTIR, UVS and HPLC were used. In the FTIR approach, approximately 200 mg of pure KBr crystal was taken and finely crushed by mortar and pestle. Then 2% of the powdered albendazole was added. The two powders were thoroughly mixed together and using oil pressure rotary pump a disk was formed. Another disk of KBr without albendazole was also prepared. Then both disks were placed on the sample handler and inserted into the instrument [13].

In the UVS spectrometric test, a portion of a clear solution of both sample and standard were taken separately. These solutions were diluted with acidified methanol to obtain solutions containing 10 μg of albendazole per mL. Then, the test and the standard solutions were examined spectrophotometrically over the spectral range from 200 to 400 nm [12].

HPLC equipped with an ultraviolet-visible detector (SPD-20A/20AV, Shimadzu Corporation, Japan) was used for qualitative and quantitative evaluation. HPLC grade solvents and albendazole 200 mg standard (USP-CRM, USA) were used. In HPLC system, we used a 254 nm detection wave length and a 4.6 mm × 25 cm column type that contains 5 μparticle sizes. According to USP [13], the flow rate was one mL per minute. The total chromatography run time was 12 min. The mobile phase in HPLC machine was prepared by dissolving 0.5 g of monobasic ammonium phosphate in 400 mL of deionized HPLC grade water and 600 mL methanol. The sample was prepared by transferring 100 mg of finely powdered tablets of albendazole into a 50 mL volumetric flask. Then, 5 mL of diluent was added, sonicated for 30 min, diluted with methanol to volume, mixed thoroughly and filtered using Whatman filter paper. The first 15 mL of the filtrate was discarded and then 5 mL of the clear stock filtrate was transferred into the second volumetric flask and diluted with methanol to obtain a solution containing 200 μg of albendazole per mL [13]. The standards were prepared based on the direction in USP [13]. Prepared solutions were passed through 0.45 μm syringe filter and transferred into 1.5 mL HPLC vial. About 20 μL of the standard and the sample were injected into the HPLC machine separately. The retention time of the peak for albendazole in the sample and the standard were compared for identification since the retention time of the peak for albendazole in the chromatogram of the sample corresponds to that of the standard preparation [13]. The peak area was used for quantification [4, 14]. All procedures were done at least three times to increase the precision. System stability was checked prior to running each sample.

## Data management and analysis

The data were checked, coded, and entered into a Microsoft excel work sheet and descriptive statistics were used to summarize the data. The mean, standard deviation, and relative standard deviation (RSD) were used to compare the albendazole standard with different brands. The amount of albendazole in each brand or tablet was calculated by considering the peak area of the sample, the standard, dilution rate and the label claim [13]. According to USP, an albendazole tablet should contain 90–110% of the labelled amount for acceptable quality [13].

## Results

All brands of albendazole tablets had uniform shape, size and colour. There were no breakage and cracking of tablets in all brands. Gross contamination was observed in one batch (Table 1).

**Table 1:** Physical characteristics of the tablets

Product	Label dose (strength) of API (mg/tablet)	Uniformity of shape	Uniformity of size	Uniformity of colour	Breaks, cracks and splits	Surface spot or contamination
Brand 1	2500	Yes	Yes	Yes	No	No
	300	Yes	Yes	Yes	No	No
Brand 2	2500	Yes	Yes	Yes	No	No
Brand 3	2500	Yes	Yes	Yes	No	No
	600	Yes	Yes	Yes	No	Yes^a^
Brand 4	600	Yes	Yes	Yes	No	No
Brand 5	2500	Yes	Yes	Yes	No	No
	300	Yes	Yes	Yes	No	No
Brand 6	2500	Yes	Yes	Yes	No	No
	300	Yes	Yes	Yes	No	No

^a^ A sample with gross contamination, *API* Active Pharmaceutical Ingredient

The packing information and labelling of the different albendazole brands were assessed based on WHO criteria [15]. Out of the 6 brands, one (16.7%) brand or two (20%) batches had no leaflet or package insert (Table 2).

**Table 2:** Packing information and label for the different brands

Product	Container and closer	Medicine strength (mg/tablet)	Dosage statement	Batch/ Lot No	Manufacture and expiry date	Storage information	Leaflet or package insert
Brand 1	Yes	Yes	Yes	Yes	Yes	Yes	Yes
	Yes	Yes	Yes	Yes	Yes	Yes	Yes
Brand 2	Yes	Yes	Yes	Yes	Yes	Yes	Yes
Brand 3	Yes	Yes	Yes	Yes	Yes	Yes	Yes
	Yes	Yes	Yes	Yes	Yes	Yes	Yes
Brand 4	Yes	Yes	Yes	Yes	Yes	Yes	Yes
Brand 5	Yes	Yes	Yes	Yes	Yes	Yes	No^a^
	Yes	Yes	Yes	Yes	Yes	Yes	No^a^
Brand 6	Yes	Yes	Yes	Yes	Yes	Yes	Yes
	Yes	Yes	Yes	Yes	Yes	Yes	Yes

^a^There was no leaflet or package insert

The weight uniformity of albendazole tablets of each brand are presented in Table 3. The study revealed that out of the ten batches, 4(40%) did not comply with the official pharmacopoeial specification limit [13].

**Table 3:** Packing information, expiration date, and weight of different brands of albendazole

Product	Label dose (strength) of API (mg/tablet)	Blisters x tablet	Expiration date	Weight mg (mean ± SD) (*n* = 20)	RSD
Brand 1	2500	55 (11 × 5)	2018	5950 ± 116.20	1.95
	300	60 (10 × 6)	2019	1980 ± 69.71	3.52^a^
Brand 2	2500	60 (15 × 4)	2019	6080 ± 55.75	0.91
Brand 3	2500	60 (12 × 5)	2020	4900 ± 201.53	4.11^a^
	600	60 (5 × 12)	2020	4980 ± 43.02	0.86
Brand 4	600	55 (11 × 5)	2019	4880 ± 60.12	1.23
Brand 5	2500	60 (12 × 5)	2019	5870 ± 56.88	0.96
	300	60 (10 × 6)	2019	1810 ± 36.63	2.02^a^
Brand 6	2500	60 (12 × 5)	2019	5730 ± 123.19	2.15^a^
	300	55 (11 × 5)	2019	4830 ± 89.35	1.85

^a^Did not meet the specification for uniformity of weight, *API* Active Pharmaceutical Ingredient, *SD* standard deviation, *RSD* Relative Standard Deviation

**Fig. 1:**
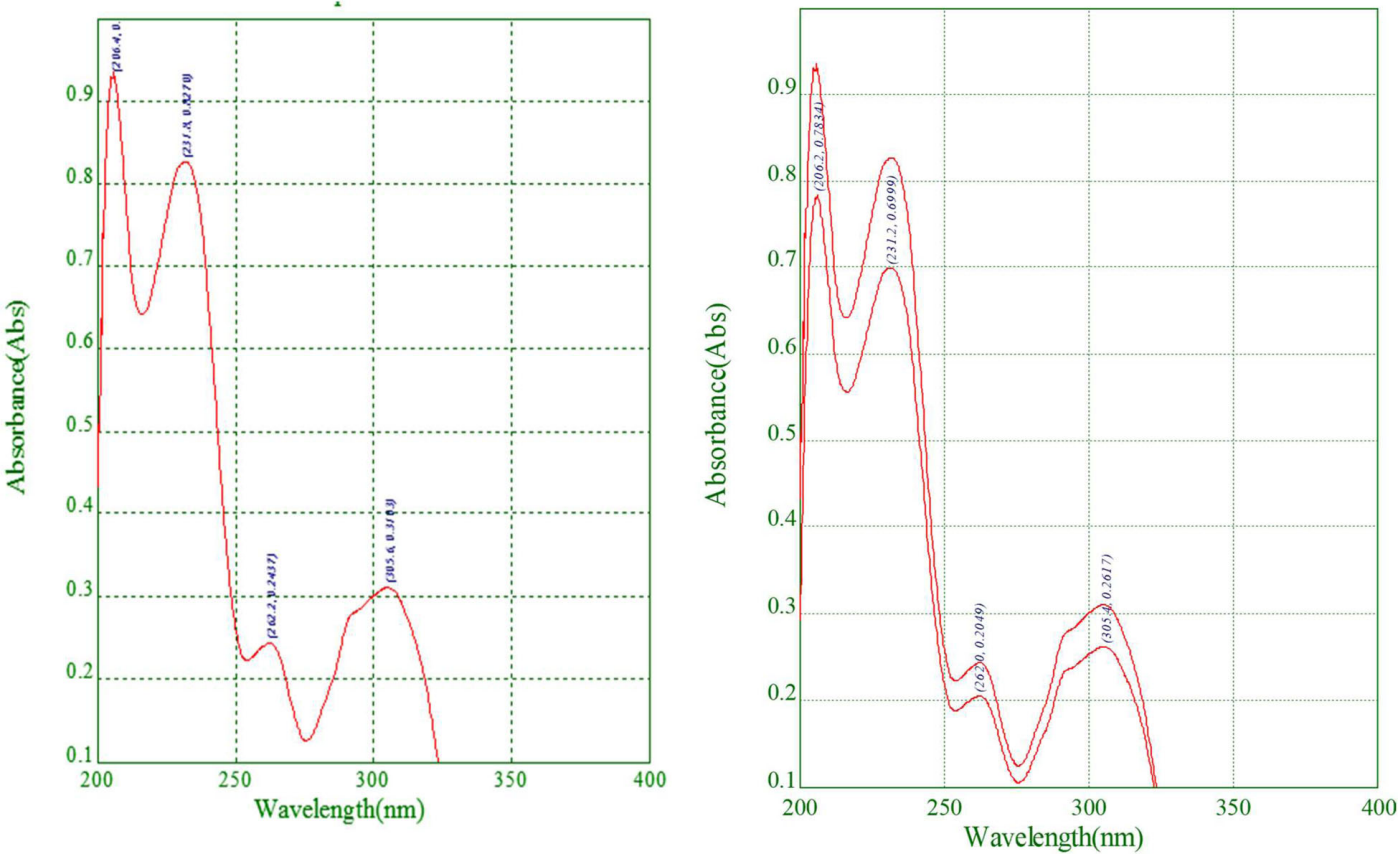
Ultraviolet-visible spectra of Albendazole for primary identification test (left-side standard and right-side sample scans, being superimposed)

**Fig. 2:**
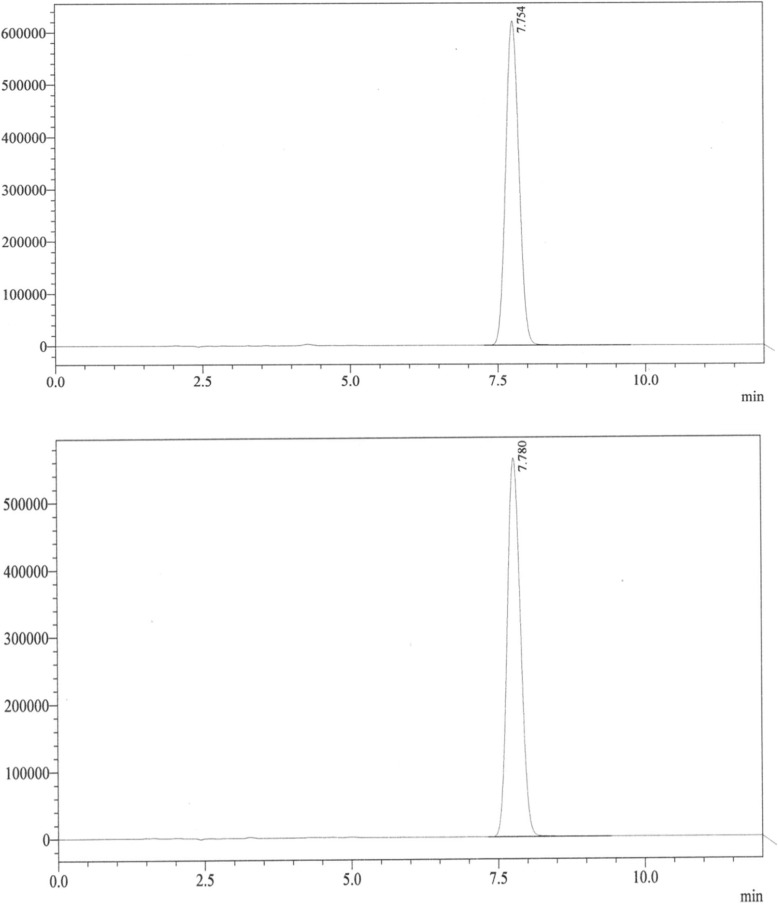
Chromatograph of standard and sample, respectively (upper standard, lower sample)

Concerning the identification test, all three methods (UVS, FTIR and HPLC) confirmed that all brands passed the identity test, verifying that all samples had albendazole as an active pharmaceutical ingredient (API) in their formulations (Table 4, Figs. 1 and 2). Fig. 1 depicts that both the sample and the standard have maximum absorbance on the same or equivalent wave length which confirmed that the sample had the intended API. Figure 2 shows the chromatographs of the standard and sample at nearly the same retention time.

**Table 4:** Different strengths of the drug that passed the test requirement

Product	Label dose (strength) of API (mg/tablet)	Identity test
Brand 1	2500	Pass
	300	Pass
Brand 2	2500	Pass
Brand 3	2500	Pass
	600	Pass
Brand 4	600	Pass
Brand 5	2500	Pass
	300	Pass
Brand 6	2500	Pass
	300	Pass

*API* Active Pharmaceutical Ingredient

As it is indicated in Tables 5, 3 (50%) brands and 3(30%) batches contained the amount of albendazole which was lower than the acceptable limit. However, the result indicated that there was no drug sample which contained above the acceptable limit.

**Table 5:** Percentage (%) and content of brands in mg

Product	Label dose (strength) of API (mg/tablet)	Label claimed (%)	Assay (% mean ± % SD)	RSD	Assay test (90–110%)
Brand 1	2500	100	94.97 ± 2.57	2.71	Pass
	300	100	97.64 ± 0.74	0.76	Pass
Brand 2	2500	100	99.88 ± 0.60	0.60	Pass
Brand 3	2500	100	105.85 ± 9.82	9.28	Pass
	600	100	100.82 ± 4.61	4.57	Pass
Brand 4	600	100	87.22^a^ ± 1.57	1.80	Fail
Brand 5	2500	100	94.93 ± 1.80	1.90	Pass
	300	100	86.92^a^ ± 1.28	1.47	Fail
Brand 6	2500	100	84.75^a^ ± 0.69	0.82	Fail
	300	100	105.73 ± 0.99	0.93	Pass

^a^Below 90% (the acceptable range is 90–110%) [12]; *SD* Standard Deviation, *RSD* Relative Standard Deviation, *API* Active Pharmaceutical Ingredient

## Discussion

For successful therapeutic effect, a pharmaceutical product should contain the appropriate amount of active pharmaceutical ingredient (API) and required physical characteristics. The manufacturer should also provide appropriate information including the product name, amount of API, the indications, contra-indications, warnings, storage information, expiration date, batch number, withdrawal periods, manufacturer name and address, and leaflet insert. In this study, problems were observed with labelling and physical characteristic by gross contamination in one batch and absence of leaflet in one brand (two batches).

Considering the weight per tablet for each brand or batch of a drug is important to assure uniformity of dosage of a drug [13]. Dosage uniformity helps to ensure a constant dose of drug between individual dosage forms. In this study, assessment of the weight uniformity revealed that four batches did not meet the criteria. In contrast to the finding of this study, absence of variation was reported by Othman [16] in Yemen. The difference may be related to the manufacturing practices.

According to the official monograph of the USP [13], the API of a drug should not be less than 90% and greater than 110% of the label claim. If the API of drug is within the acceptance range, the drug can produce the required therapeutic effects with limited side effects on the patient. If the drug contains higher than the expected amount, it may not be safe. On the other hand, if the drug has lower amount of API, it may not cure the animal and my favour the development of anthelmintic drug resistance. There are frequent reports that show resistance to common anthelmintic drugs especially in warm and humid parts of the world [17].

In this study, out of the ten batches, 3(30%) had less than 90% of the API. This confirmed the presence of sub-therapeutic doses of anthelmintic drugs in the legal market of the country. It is obvious that the occurrence of such a scenario might not cure the patient or favour the development of resistance [18, 19]. The substandard products could originate from poor preparation techniques during formulation and subsequent manufacturing processes, incorrect weighing and mixing, or it may be intentional to reduce the cost of production [20]. A relatively high prevalence of poor quality of albendazole was also reported in a separate study by Suleman et al. [21] in Ethiopia. In Yemen, Othman [16] assessed the quality of seven brands of albendazole and found that only two brands fulfil the British Pharmacopeia quality control standards. The presence of counterfeit anthelminthic drugs was also reported by Khan et al. [8] in Cambodia.

Despite some controlling practices in Ethiopia, there are still practices of misuse and smuggling of anthelmintic drugs. In addition, no strategy is in use to preserve and maintain the efficacy of anthelmintic drugs or to delay and prevent the emergence of anthelmintic drug resistance [22]. The findings of this study raise an alarming concern with regards to suboptimal drugs circulating in the legal market. This can contribute towards several interrelated problems. The implications of these can be: (1) inability to achieve the therapeutic goal which compromises the welfare and productivity of the animals, (2) obligatory repetitive treatments which incur additional costs for the farmer and (3) as a long-term effect, it may increase the presence of resistant parasite population. All of the aforementioned consequences of sub-standard drugs circulating in the legal market may further aggravate the already existing anthelmintic resistance and poor efficacy scenarios reported from different parts of the country [2, 7]. The clinical significance of drug resistance is highly crucial in notorious parasites like *H. contortus* in which a massive infection can kill the host [3, 7, 23].

## Conclusions

There were variations in contents of samples of different brands of albendazole. This variation can have significant influence on the drug activity. The presence of low level of API below the official recommended limit often results in treatment failure and favours the development of parasites resistance to the specific anthelmintic drug. Therefore, there should be a strong pre-registration evaluation and regular monitoring of the quality of drugs circulating in the market.

## Additional files

Additional file 1: Chromatogram of samples and the standard. (DOC 303 kb)

Additional file 2: UvVis readings of Albendazole Reference and sample. (DOCX 62 kb)

## Abbreviations

API: Active Pharmaceutical Ingredient
FTIR: Fourier Transformed Infrared Spectroscopy
HPLC: High Performance Liquid Chromatography
RSD: Relative Standard Deviation
SD: Standard Deviation
USP: United States Pharmacopeia
UVS: Ultraviolet Visible Spectroscopy
WHO: World Health Organization
