# Supplementary figures and images for: Quality of albendazole tablets legally circulating in the pharmaceutical market of Addis Ababa, Ethiopia: physicochemical evaluation
Source: BMC Pharmacol Toxicol. 2019 Apr 25;20:20. doi: 10.1186/s40360-019-0299-5 (PMC6485143; doi:10.1186/s40360-019-0299-5)

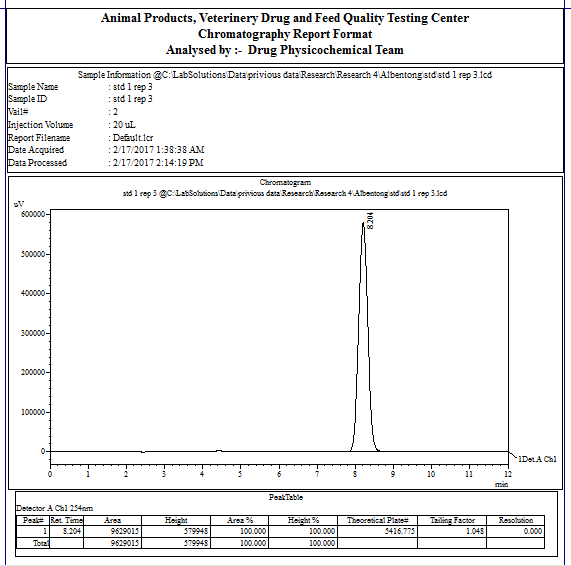
Standard


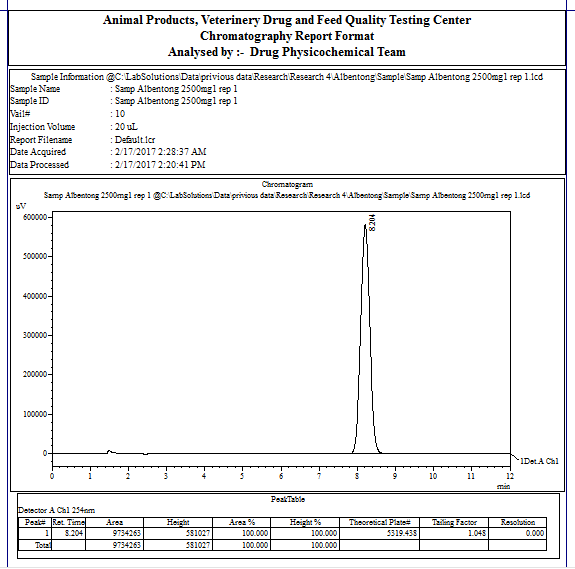
Sample


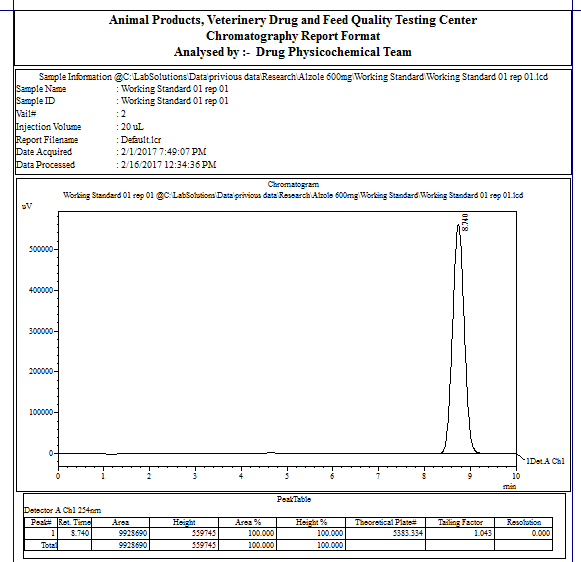
Standard


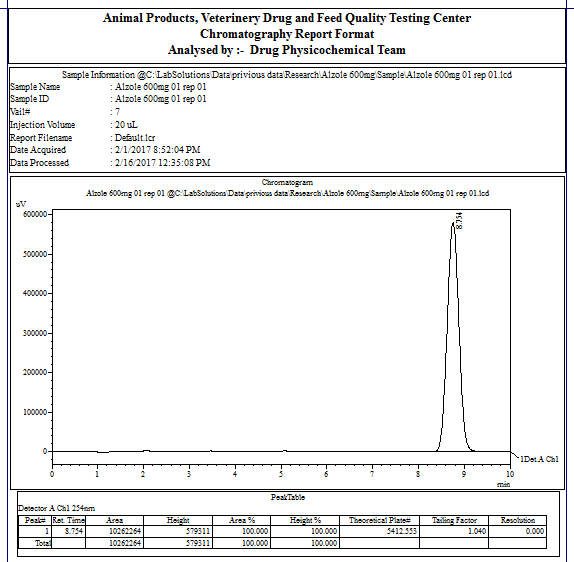
Sample 600mg


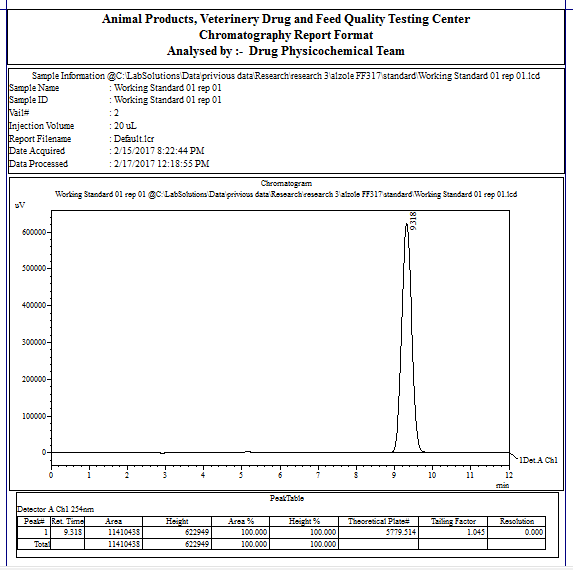
Standard


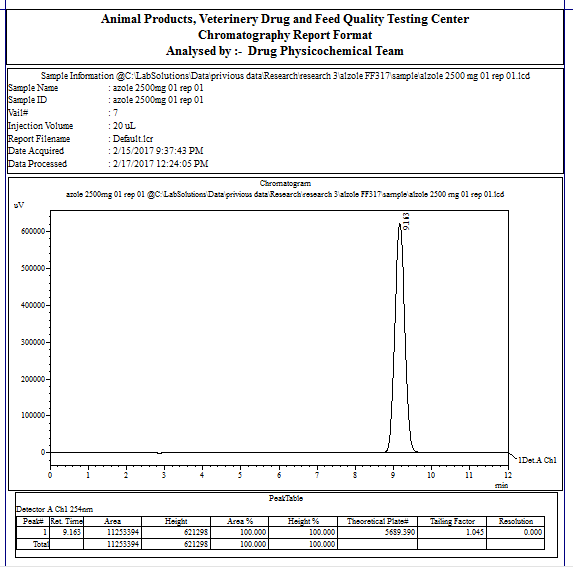
 sample 2500mg


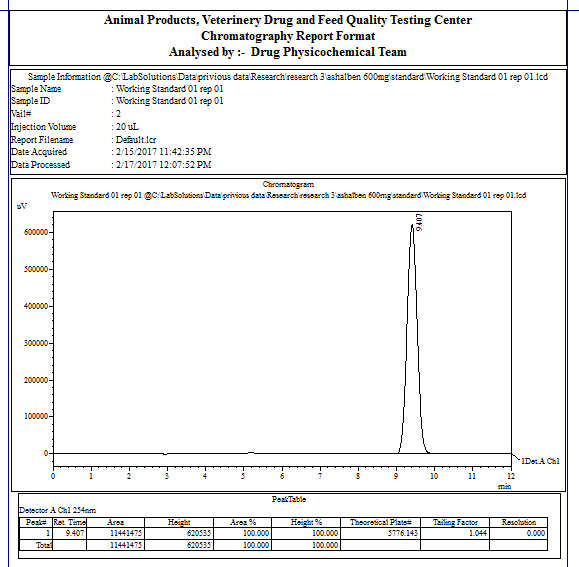
Standard


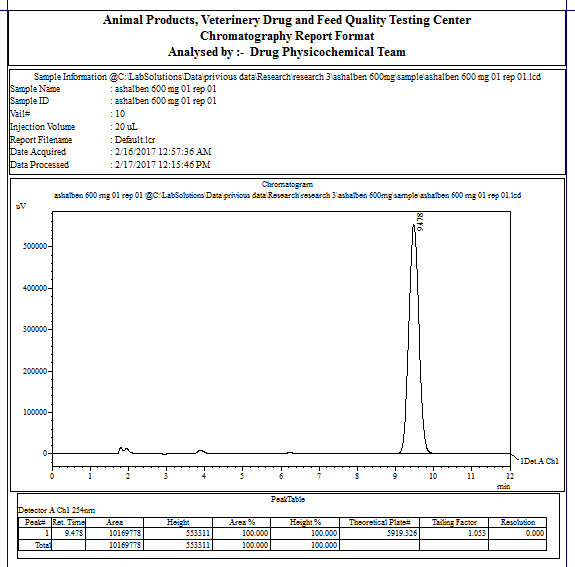
Sample


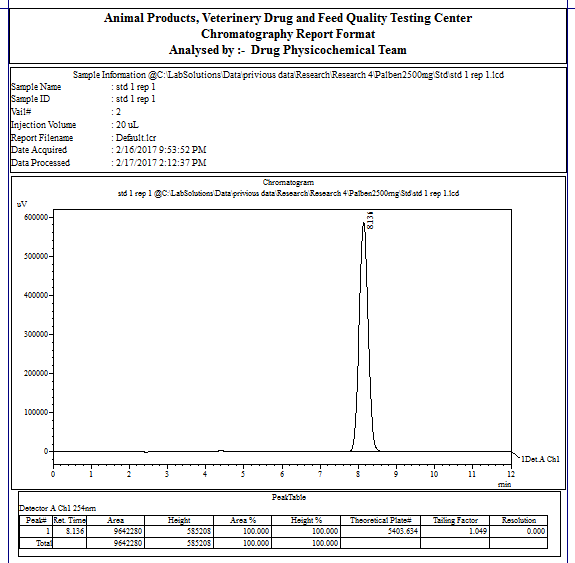
Standard


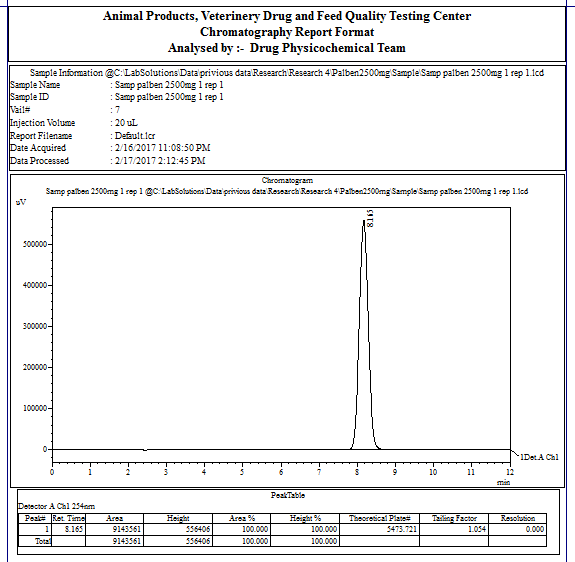
Sample

Supplement: Supplementary file 1 — Chromatogram of samples and the standard. (DOC 303 kb) [file 40360_2019_299_MOESM1_ESM.doc]
