# Supplementary material for: Quality of albendazole tablets legally circulating in the pharmaceutical market of Addis Ababa, Ethiopia: physicochemical evaluation
Source: BMC Pharmacol Toxicol. 2019 Apr 25;20:20. doi: 10.1186/s40360-019-0299-5 (PMC6485143; doi:10.1186/s40360-019-0299-5)
Supplement: Supplementary file 2 — UvVis readings of Albendazole Reference and sample. (DOCX 62 kb) [file 40360_2019_299_MOESM2_ESM.docx]

**Albendazole Reference standard and samples reading by UV-Vis spectrometry**


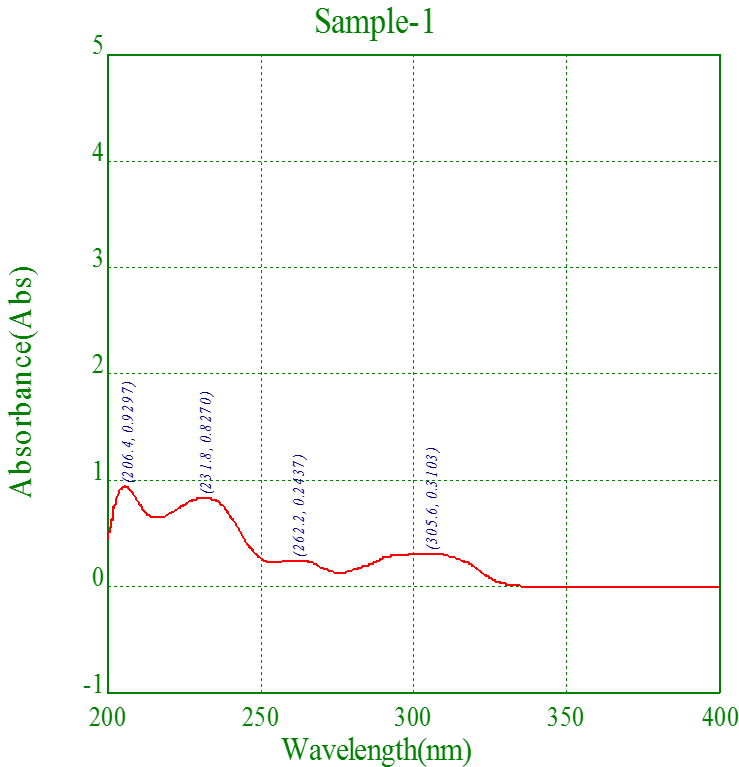


- Standard (red line), sample 1(blue line), sample 2 (green line), and sample 3 (black line), respectively
- This procedure was used for identity test only to determine either the presence or absence of active pharmaceutical ingredient
